# CMR-based cardiac phenotyping in different forms of heart failure

**DOI:** 10.1007/s10554-024-03145-4

**Published:** 2024-06-15

**Authors:** Torben Lange, Sören J. Backhaus, Alexander Schulz, Djawid Hashemi, Ruben Evertz, Johannes T. Kowallick, Gerd Hasenfuß, Sebastian Kelle, Andreas Schuster

**Affiliations:** 1 Department of Cardiology and Pneumology, University Medical Center Göttingen, Georg-August University, Göttingen, Germany; 2 German Centre of Cardiovascular Research (DZHK), partner site Lower Saxony, Göttingen, Germany; 3 Department of Cardiology, Campus Kerckhoff of the Justus-Liebig-Universität Gießen, Kerckhoff-Clinic, Bad Nauheim, Germany; 4 Department of Internal Medicine/Cardiology, Charité Campus Virchow Clinic, Berlin, Germany; 5 German Centre for Cardiovascular Research (DZHK), partner site Berlin, Berlin, Germany; 6 Institute for Diagnostic and Interventional Radiology, University Medical Center Göttingen, Georg-August University, Göttingen, Germany

**Keywords:** CMR imaging, CMR feature-tracking, Heart failure, Myocardial strain assessment, Left atrioventricular coupling index, CMR-based tissue characterization

## Abstract

Heart failure (HF) is a heterogenous disease requiring precise diagnostics and knowledge of pathophysiological processes. Since structural and functional imaging data are scarce we hypothesized that cardiac magnetic resonance (CMR)-based analyses would provide accurate characterization and mechanistic insights into different HF groups comprising preserved (HFpEF), mid-range (HFmrEF) and reduced ejection fraction (HFrEF). 22 HFpEF, 17 HFmrEF and 15 HFrEF patients as well as 19 healthy volunteers were included. CMR image assessment contained left atrial (LA) and left ventricular (LV) volumetric evaluation as well as left atrioventricular coupling index (LACI). Furthermore, CMR feature-tracking included LV and LA strain in terms of reservoir (Es), conduit (Ee) and active boosterpump (Ea) function. CMR-based tissue characterization comprised T1 mapping as well as late-gadolinium enhancement (LGE) analyses. HFpEF patients showed predominant atrial impairment (Es 20.8%vs.25.4%, p = 0.02 and Ee 8.3%vs.13.5%, p = 0.001) and increased LACI compared to healthy controls (14.5%vs.23.3%, p = 0.004). Patients with HFmrEF showed LV enlargement but mostly preserved LA function with a compensatory increase in LA boosterpump (LA Ea: 15.0%, p = 0.049). In HFrEF LA and LV functional impairment was documented (Es: 14.2%, Ee: 5.4% p < 0.001 respectively; Ea: 8.8%, p = 0.02). This was paralleled by non-invasively assessed progressive fibrosis (T1 mapping and LGE; HFrEF > HFmrEF > HFpEF). CMR-imaging reveals insights into HF phenotypes with mainly atrial affection in HFpEF, ventricular affection with atrial compensation in HFmrEF and global impairment in HFrEF paralleled by progressive LV fibrosis. These data suggest a necessity for a personalized HF management based on imaging findings for future optimized patient management.

## Introduction

Heart failure (HF) presents a major socioeconomic challenge in cardiovascular medicine and correlates with a notable risk for hospitalization and adverse outcome [1]. Accurate imaging and assessment of cardiac function are essential for optimal risk stratification, diagnosis and management of HF. Currently, left ventricular ejection fraction (LVEF) stands as the predominant clinical parameter for phenotyping and distinguishing various forms of HF [2]. These classifications encompass HF with preserved ejection fraction (HFpEF), mildly reduced ejection fraction (HFmrEF) and reduced ejection fraction (HFrEF) [3]. However, relying solely on LVEF has evident limitations for comprehensive myocardial performance evaluation as preserved LVEF may not indicate preserved myocardial function [4]. In this context, cardiac magnetic resonance (CMR) imaging has been established as an appropriate and superior technique to comprehensively depict and assess multifaceted pathomechanisms across cardiac chambers in HF. With growing evidence of CMR feature tracking (CMR FT)-derived strain analyses outperforming LVEF by providing incremental diagnostic and prognostic characteristics, cardiovascular imaging specialists increasingly apply CMR FT based deformation assessments for the evaluation of HF [5–7]. Moreover, attention to atrial function signifies a significant advancement in cardiac performance analysis, providing crucial prognostic value often overlooked in traditional cardiac assessments [8–10]. Combining CMR-based atrial and ventricular volumetric parameters including left atrioventricular coupling index (LACI) allows for a more precise and holistic cardiac performance assessment with considerable prognostic implications beyond functional assessment of individual cardiac chambers [11, 12]. Additionally, quantitative cardiac evaluations represent another crucial aspect of comprehensive CMR image analyses, offering insights into myocardial tissue composition with significant prognostic implications [13, 14]. Notably, (diffuse) myocardial fibrosis emerges a key pathophysiological mechanism in HF, that can be non-invasively assessed by CMR imaging using T1 mapping techniques and late-gadolinium enhancement (LGE) analyses [15]. Adverse remodeling, increased wall stiffness and associations with poor outcome underscore the significant role of myocardial fibrosis in HF assessment. [16] .

However, detailed multi-parametric analyses of myocardial pathophysiological contributions particularly encompassing the LA-LV interplay within the HF evolution and continuum remain scarce. Therefore, the aim of the current study was to address this critical gap by providing a comprehensive multi-parametric CMR-derived analysis of myocardial contributions across different HF types, thereby paving the way for improved diagnostic and therapeutic strategies across the HF spectrum.

## Methods

### Study population

54 patients with chronic HF as well as 19 healthy volunteers were enrolled in this study as previously published [17–22]. In brief, HF patients were classified into different subgroups according to current guideline recommendations [2]:

1. HFpEF (LVEF ≥ 50%, but E/e´ ≥ 13 and mean septal e´ and lateral wall < 9cm/s or LAVI > 34 ml/m^2^ **and** plasma levels of NT-proBNP > 125 pg/mL
2. HFmrEF (LVEF = 40 – 49%) and presence of additional criteria as per [1]
3. HFrEF (LVEF < 40%)

Exclusion criteria comprised severe valvular disease, uncontrolled hypertension and atrial fibrillation at enrollment. Participants underwent transthoracic echocardiography, CMR imaging, laboratory testing and clinical examination Moreover, a clinical HF assessment was performed, comprising a documentation of NYHA status, Borg score, 6-min-walking-test (6mwt) and fatigue as well as edema evaluation. In addition, a fourth group of age- and sex–matched volunteers was recruited undergoing an identical protocol.

The study was approved by the local Ethics Committee of the Charité-Universitätsmedizin Berlin, complied with the Declaration of Helsinki and was registered at the German Register for Clinical Studies (DRKS, registration number: DRKS00015615). All patients gave written informed consent before participation.

### CMR image analyses

CMR imaging was performed on a 1.5 Tesla scanner (Achieva, Philips Healthcare, Best, The Netherlands) with a 5-channel cardiac surface coil in the supine position in all patients as previously described [17]. Identical scan protocols included ECG-gated balanced steady-state free precession (bSSFP) images in 2- and 4-chamber orientation with multiple breath-holds at end-expiration. Typical scan parameters for bSSFP images were: TR 3.3 ms, TE 1.6 ms, 60° flip angle, voxel size = 1.8 × 1.7 × 8.0 mm^3^ with 50 phases per cardiac cycle. Additional T1-mapping was performed using a modified Look-Locker (MOLLI) 5s(3s)3s-scheme. Extracellular volume (ECV) maps were generated from pre- and post-contrast T1-maps as recently published [23]. A black-blood prepared, T1-weighted, spoiled-gradient, multi-echo sequence was applied for modified DIXON-imaging (mDIXON) to visually assess LGE.

**Fig. 1:**
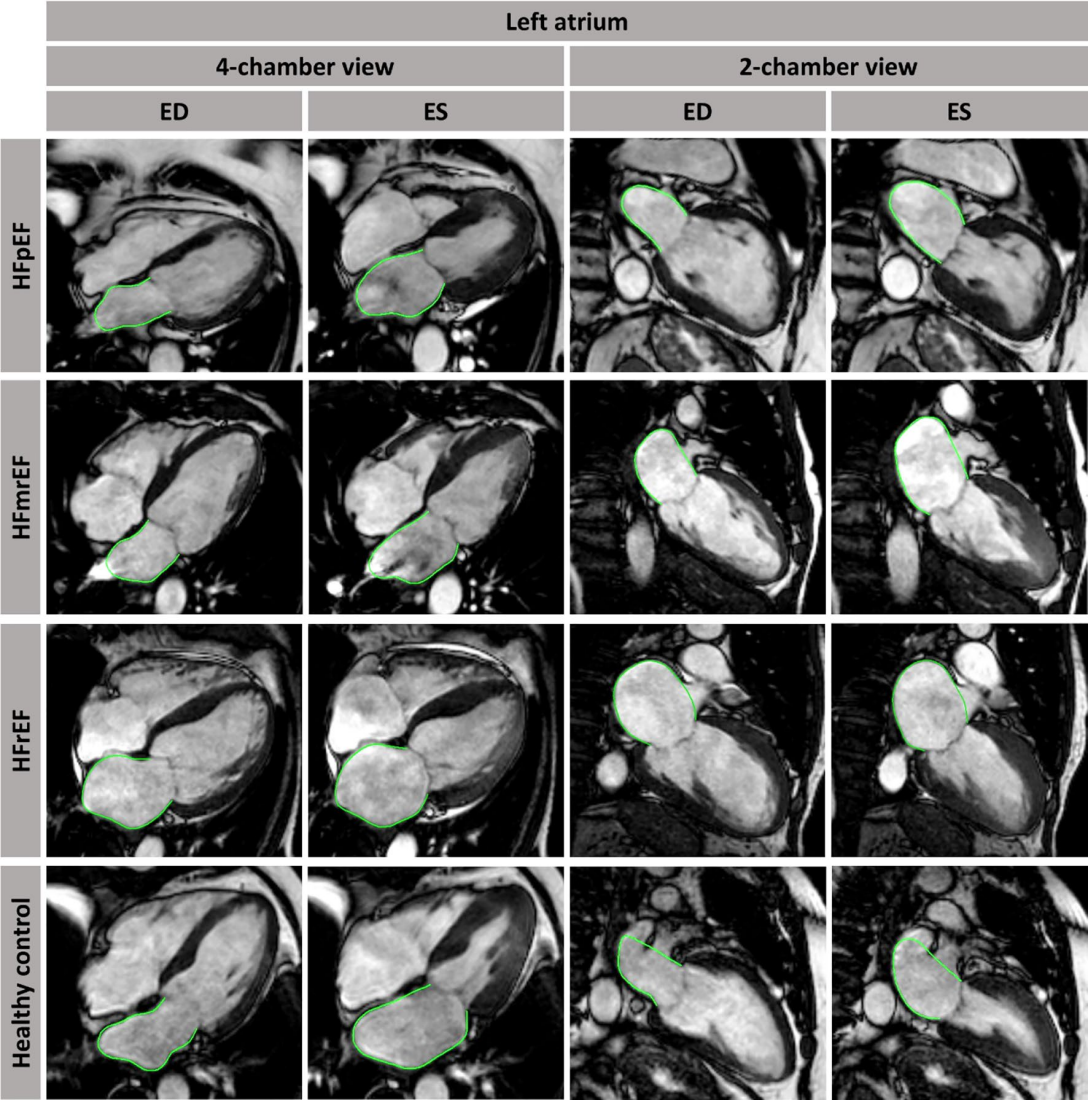
CMR-Feature-Tracking of the left atrium in long-axis 2- and 4-chamber views in enddiastole (ED) and endsystole (ES). Analyses were performed in patients with heart failure with preserved (HFpEF), mid-range (HFmrEF) and reduced ejection fraction (HFrEF) as well as in healthy controls

CMR-based volumetric assessments included left atrial (LA) and left ventricular (LV) end-diastolic volumes (EDV) as well as end-systolic volumes (ESV) using dedicated post-processing software (Qmass, Medis Medical Imaging Systems, Leiden, The Netherlands). Left atrioventricular coupling index (LACI) was calculated as ratio between LA EDV and LV EDV and expressed as a percentage [24]. LA and LV volumes were measured in the same end-diastolic phase. Strain values were analyzed using established and validated Feature-Tracking FT software (TomTec Imaging Systems, Unterschleissheim, Germany) as previously described and an average from three measurements was calculated [8, 25] (Fig. 1).

**Fig. 2:**
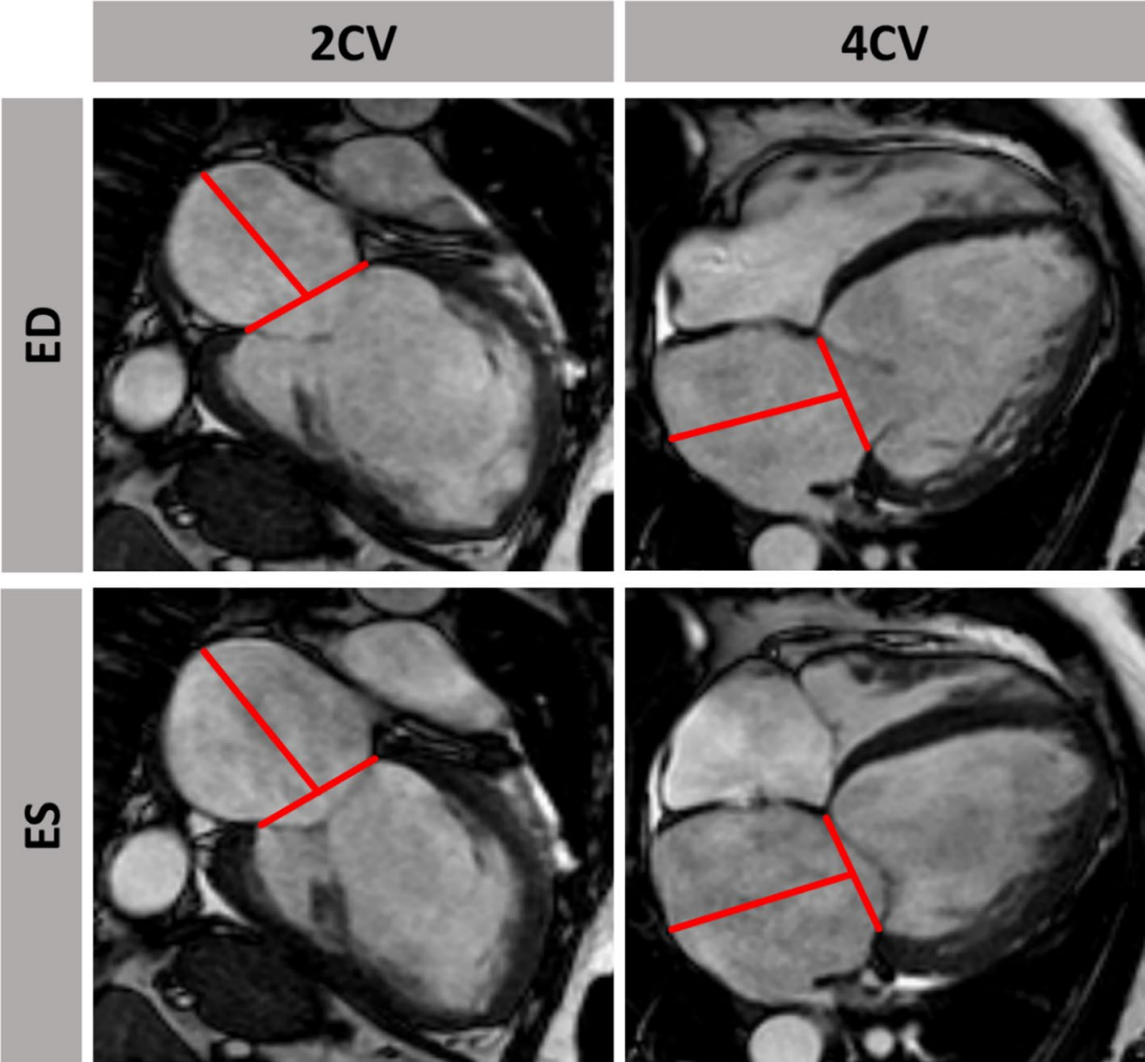
Illustration of left atrial long axis strain (LA LAS) in long-axis 2- and 4- chamber view (CV) at left ventricular end-diastole (ED) and end-systole (ES). LA LAS was analyzed as previously described [26] assessed between the middle of a line connecting the origins of the mitral leaflets and a line linking the LA posterior portion of the greatest distance in regard to the middle of the mitral reference line

**Fig. 3:**
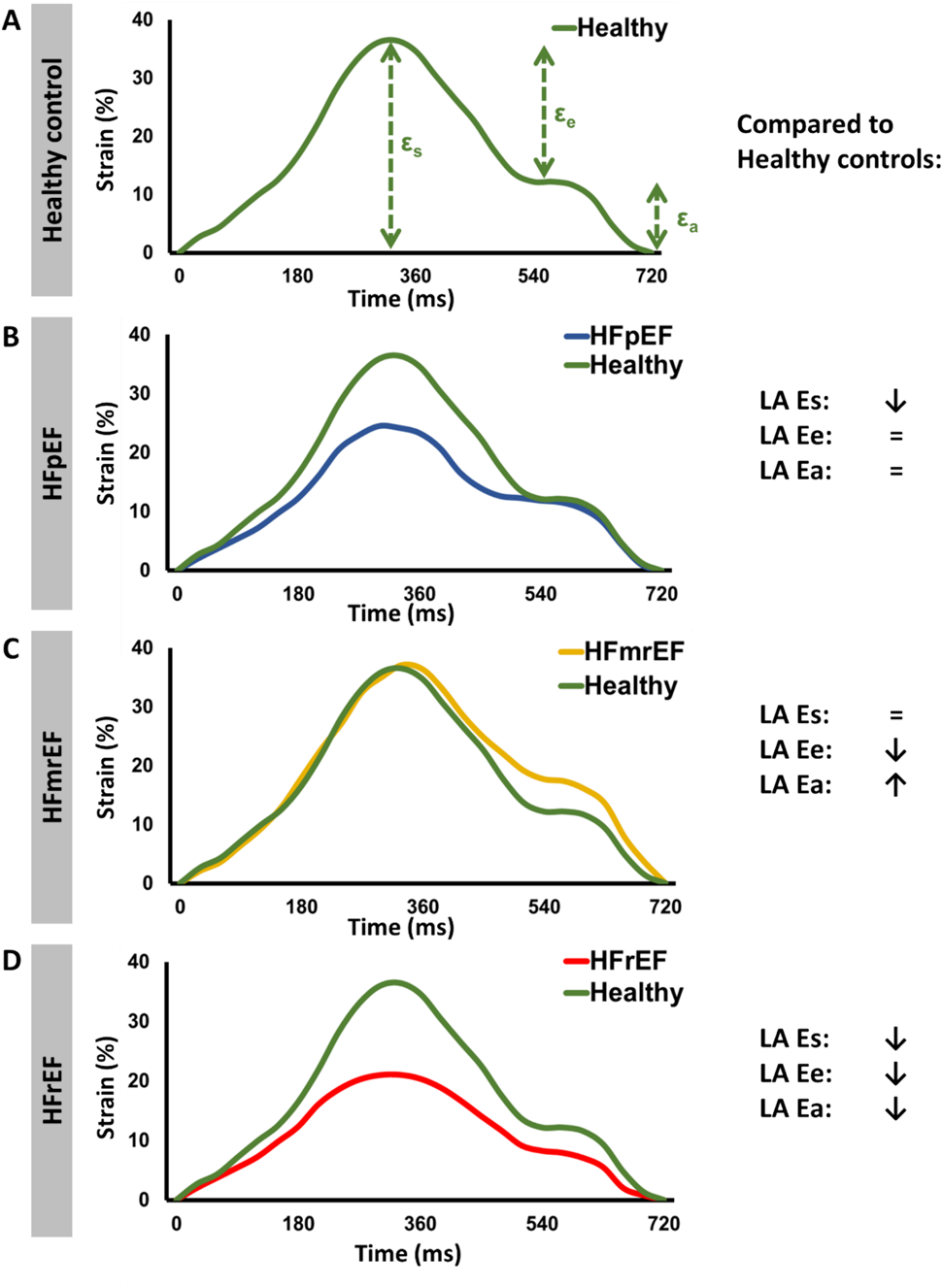
Left atrial (LA) strain (E) analyses in healthy controls and across patients with heart failure with preserved (HFpEF), mid-range (HFmrEF) and reduced ejection fraction (HFrEF). Es: reservoir function, Ee: conduit function, Ea: boosterpump function

LA long axis strain (LAS) was analyzed as previously described [26]: Initially, a line connecting the origins of the mitral leaflets was set. Subsequently, a line connecting to the LA posterior portion of the greatest distance in regard to the reference line was plotted (Fig. 2). CMR-FT-derived LA strain values were divided into three functional components: 1) total strain (Es) corresponding to atrial reservoir function describing the collection of venous return during ventricular systole, 2) passive strain (Ee) corresponding to atrial conduit function representing blood flow to the ventricle during early ventricular diastole and 3) active strain (Ea) reflecting atrial booster pump function as an amplification of ventricular filling during late diastole (Fig. 3 A)[27].

### Statistical analyses

Categorical variables are reported in absolute numbers and percent values. Normal distribution of continuous parameters was tested using the Shapiro–Wilk test and variables are expressed using median and interquartile range. In each HF cohort median values for functional CMR parameters were calculated to compare clinical values between these subgroups. Continuous variables were compared using the Mann–Whitney U test. For the assessment of correlations according to a case–control principle including both healthy controls and the respective HF subgroup, Spearman’s rank correlation coefficient was used for non-normally distributed data and Pearson´s correlation when data were normally distributed. Provided p-values are two-sided and were considered significant ≤ 0.05. All statistical analyses were performed using IBM SPSS Statistics Software Version 28 for Windows 10 (IBM, Armonk, NY, USA).

## Results

### Study population

Baseline characteristics of HF patients as well as of the healthy control group have been published previously [17–22]. CMR imaging and analyses were performed in 22 patients diagnosed with HFpEF, 17 patients with HFmrEF, 15 with HFrEF as well as in 19 healthy volunteers. In short, patients suffering HFpEF and HFmrEF were older than HFrEF and healthy control cohorts (p < 0.001 and p = 0.025). There were no relevant differences in sex between all groups. Cardiovascular risk factors did not differ amongst HF patient groups but were more frequently present in HF compared to healthy controls. HF patients had significantly more severe breathlessness (NYHA class), reduced performance in 6mwt and increased blood levels of NT-proBNP (p < 0.001) compared to the healthy control group. Coronary artery disease (CAD) was a common comorbidity and distributed as follows: HFpEF: 15/22 (68%); HFmrEF: 15/17 (88%), HFrEF: 10/15 (67%); controls: 0/18 (0%). A detailed overview of CMR imaging parameters, that have been partially reported elsewhere [17–22], is shown in Table 1.

**Table 1:** CMR imaging parameters. Data are presented as median (interquartile range). For comparison of patients and healthy control p-values were calculated. Bold numbers indicate a statistically significant difference. Mann–Whitney U test was used for testing continuous variables. ECV: extracellular volume fraction, EDVi: enddiastolic volume index, ESVi: endsystolic volume index, Es: reservoir function, Ee: conduit function, Ea: boosterpump, GCS: global circumferential strain, GLS: global longitudinal strain, LA: left atrial, LV: left ventricular, LAEF: left atrial ejection fraction, LACI: left atrioventricular coupling index, LGE: late-gadolinium enhancement, LVEF: left ventricular ejection fraction, SVi: stroke volume index.

						p-values										
**Variables**	**HFpEF** **(n = 22)**	**HFmrEF** **(n = 17)**	**HFrEF** **(n = 15)**		**Healthy control** **(n = 19)**	HFpEF vs HFmrEF	HFpEF vs. HFrEF	HFmrEF vs HFrEF				HFpEF vs Healthy		HFmrEF vs Healthy	HFrEF vs Healthy	
Volumes																
LV Mass	49.6 (44.8–62.5)	55.2 (49.2–72.7)	74.1 (54.9–99.4)		48.0 (42.1–52.4)	0.3	**0.004**	**0.037**			0.27			**0.009**		** < 0.001**
LVEF (%)	60.1 (52.4–63.8)	45.6 (42.5–47.8)	33.9 (28.8–37.7)		65.1 (58.7–71.7)	** < 0.001**	** < 0.001**	** < 0.001**			**0.008**			** < 0.001**		** < 0.001**
LV EDVi (ml/m^2^)	70.4 (57.6–90.1)	82.5 (73.8–90.6)	114.4 (99.0–152.3)		73.2 (61.8–82.8)	0.13	** < 0.001**	** < 0.001**			1.0			**0.049**		** < 0.001**
LV ESVi (ml/m^2^)	29.2 (24.3–40.2)	44.9 (37.8–49.2)	71.3 (63.8–103.6)		25.1 (19.7–29.5)	**0.002**	** < 0.001**	** < 0.001**			0.11			** < 0.001**		** < 0.001**
LV SVi (ml/m^2^)	44.1 (35.6–48.2)	37.7 (33.1–41.6)	40.4 (34.4–48.5)		44.3 (40.5–53.5)	0.07	0.4	0.28			0.15			** < 0.001**		0.051
LAEF (%)	49.9 (35.6–54.5)	52.3 (46.8–60.1)	44.2 (28.2–51.3)		58.0 (51.5–61.7)	0.13	0.2	**0.014**			**0.004**			0.29		** < 0.001**
LA ESVi (ml/m^2^)	33.6 (24.4–41.5)	28.9 (20.9–38.3)	35.7 (27.4–52.4)		25.8 (23.5–28.4)	0.36	0.49	0.17			**0.02**			0.11		**0.002**
LA EDVi (ml/m^2^)	19.4 (12.1–23.9)	14.2 (10.1–19.3)	20.4 (13.0–34.1)		11.1 (9.1–12.8)	0.13	0.35	**0.04**			**0.002**			0.15		** < 0.001**
LA SVi (ml/m^2^)	13.8 (10.8–20.4)	14.3 (11.9–18.6)	14.3 (12.5–16.8)		14.4 (12.4–16.4)	0.92	0.96	0.97			0.86			0.73		0.66
LACI (%)	23.3 (15.3–30.3)	17.4 (13.1–23.1)	15.3 (8.8–34.1)		14.5 (12.0–19.3)	0.07	0.33	0.68			**0.004**			0.55		0.52
Strain																
LA Es (%)	20.8 (7.8)	22.3 (5.1)	14.2 (3.8)	25.4 (6.5)		0.17	**0.002**	** < 0.001**		**0.02**			0.21			** < 0.001**
LA Ee (%)	8.3 (4.0)	7.4 (3.2)	5.4 (2.7)	13.5 (4.6)		0.69	**0.03**	0.09		**0.001**			** < 0.001**			** < 0.001**
LA Ea (%)	12.5 (6.0)	15.0 (5.2)	8.8 (3.2)	12.0 (4.3)		0.05	0.05	**0.001**		1.0			**0.049**			**0.02**
LA LAS (%)	17.0 (5.4)	22.8 (6.5)	13.5 (5.7)	26.2 (7.2)		**0.006**	0.09	** < 0.001**		** < 0.001**			0.2			** < 0.001**
LV GLS (%)	− 15.6 (5.0)	− 12.6 (1.6)	− 9.0 (3.3)	-17.4 (4.1)		0.06	**0.001**	**0.01**		0.58			**0.002**			** < 0.001**
LV GCS (%)	− 26.8 (6.0)	− 18.3 (6.1)	− 13.6 (2.8)	-31.0 (5.8)		** < 0.001**	** < 0.001**	**0.03**		0.09			** < 0.001**			** < 0.001**
Tissue																
ECV total (%)	28.3 (25.7–29.8)	28.0 (26.9–30.7)	30.0 (27.1–31.3)		/	0.5	0.21	0.63	/				/			/
T1 native (ms)	988 (959–1033)	1012 (979–1039)	1038 (992–1057)		973 (948–985)	0.35	**0.046**	0.22	0.08				**0.003**			** < 0.001**
Any LGE n/N (%)	15/21 (71)	13/16 (81)	13/15 (87)		/	0.62	0.45	0.8	/				**/**			**/**
Transmural LGE n/N (%)	9/21 (43)	7/16 (44)	8/15 (53)		/	0.96	0.61	0.65	/				**/**			**/**

### Heart failure with preserved ejection fraction

There was no statistical difference of LV volumes compared to healthy control group (LV EDV: p = 1.0, LV ESV: 0.11), in contrast LA volumes were significantly increased in HFpEF patients (LA EDVi: p = 0.002, LA ESVi: p = 0.02). LACI was significantly increased compared to the control group (p = 0.004) while LAEF was significantly reduced (p = 0.004). 

HFpEF patients had significantly lower LA LAS (p = 0.006), LA reservoir and conduit strain values (p = 0.021 and p = 0.001) while LA boosterpump did not differ compared to the control group (Table 1, Fig. 3 B). Both LV GLS and GCS values showed no statistically significant difference between HFpEF and control group (GLS: p = 0.58; GCS p = 0.09). 

There was no statistically significant difference of T1 native in HFpEF compared to HFmrEF (988 ms vs. 1012 ms, p = 0.35) or healthy controls (988 ms vs. 973 ms, p = 0.08).

NT-proBNP correlated significantly with LA Ee (r = -0.41, p = 0.008), LV EDV (r = -0.45, p = 0.02) and LACI (r = 0.47, p = 0.003) in HFpEF patients. 6mwt showed significant associations with LA Ee (r = 0.47, p = 0.002) LACI (r = -0.41, p = 0.01), LV EDV (r = 0.54, p = 0.006) and LV ESV (r = 0.42, p = 0.036).

### Heart failure with mildly reduced ejection fraction

**Fig. 4:**
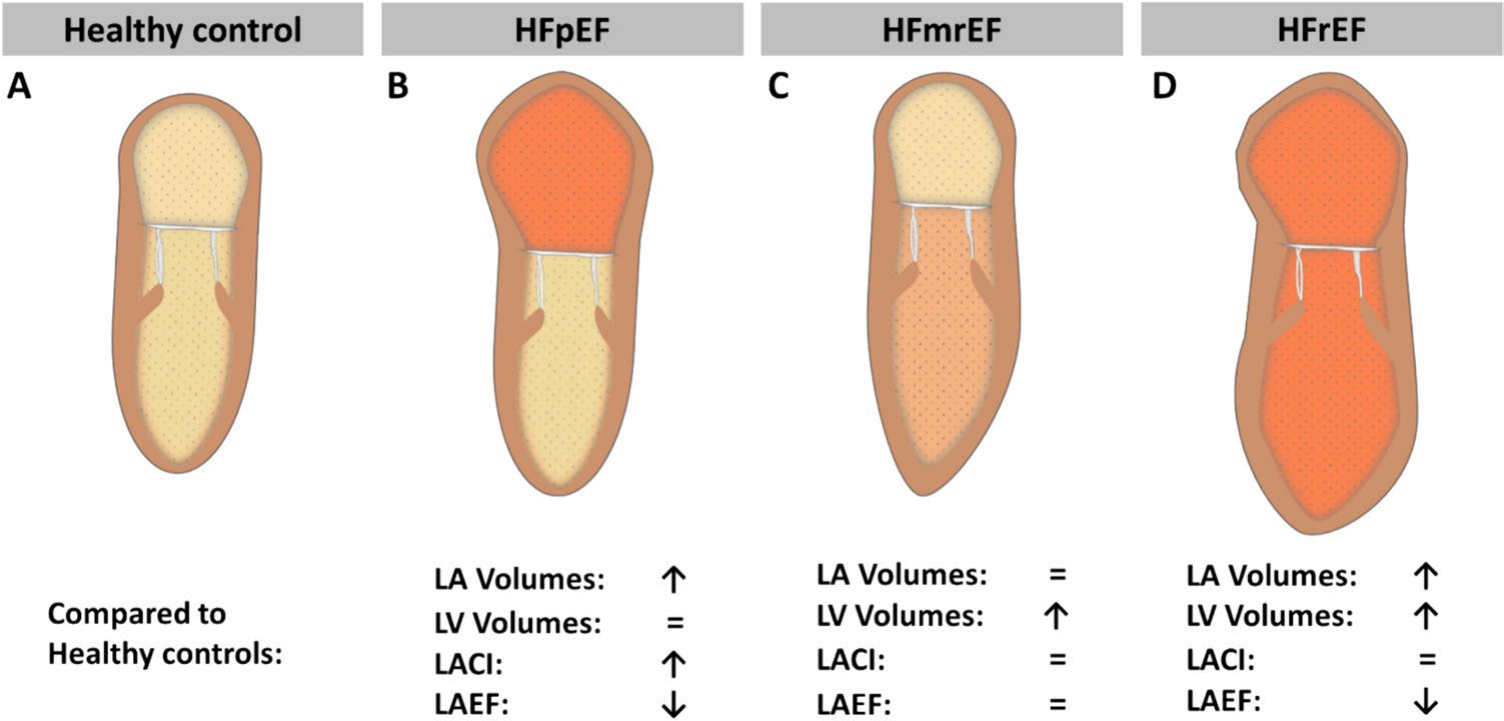
Volumetric analyses in heart failure. Left atrial (LA) and left ventricular (LV) volumetric analyses in healthy controls and across patients with heart failure with preserved (HFpEF), mid-range (HFmrEF) and reduced ejection fraction (HFrEF). LAEF: left atrial ejection fraction, LACI: left atrioventricular coupling index

In patients with HFmrEF, only LV volumes were increased compared to healthy controls (LV EDV: p = 0.049 and LV ESV: p < 0.001), while LA volumes, LAEF and LACI did not differ (Fig. 4 C).

There was no statistical difference between LA LAS and LA reservoir strain compared to the healthy control group (LA LAS: p = 0.2 and LA Es: p = 0.21). In contrast, boosterpump strain was significantly higher than in healthy subjects (15.0% vs. 12.0%, p = 0.049), while LA conduit function was reduced (7.4% vs. 13.5%, p < 0.001) (Fig. 3 C).

T1 native was significantly higher in HFmrEF compared to healthy controls (1012 ms vs. 973 ms, p = 0.003). NT-proBNP correlated with atrial parameters (LA Ee [r = -0.6, p < 0.001] as well as ventricular values (LV ESV [r = 0.53, p < 0.001], LV GLS [r = 0.4, p = 0.05] and LV GCS [r = 0.52, p = 0.007]). Furthermore, 6mwt showed associations with LA Ee (r = 0.51, p = 0.002) and LA Ea (r = -0.35, p 0.038).

### Heart failure with reduced ejection fraction

In HFrEF patients all LA and LV volumes including LV mass were significantly increased compared to healthy controls (Fig. 4 D). While there was no significant difference in LACI, LAEF was significantly reduced (p < 0.001). Furthermore, all LA strain parameters were significantly lower than in healthy subjects (p < 0.001 for LA LAS, LA Es and LA Ee, respectively and LA Ea: p = 0.02) (Fig. 3 D).

Amongst all patients with an impaired systolic LV function ventricular GLS and GCS were progressively impaired with increasing severity of HF and significantly lower than in healthy controls (Table 1).

Native T1 was significantly higher in HFrEF patients compared to HFpEF (1038 ms vs. 988 ms, p = 0.046) and healthy controls (1038 ms vs. 973 ms, p < 0.001). Although there was no statistical difference for ECV and the presence of LGE between the HF subgroups, values and percentage amount at least numerically increased with advancing stages of HF.

NT-proBNP was significantly associated with both atrial (LA Es [r = -0.67, p < 0.001], LA Ee [r = -0.71, p < 0.001], LA Ea [r = -0.36, p = 0.04] and ventricular functional parameters (LV GLS [r = 0.63, p < 0.001], LV GCS [r = 0.72, p < 0.001], LV EDVi [r = 0.57, p < 0.001] and LV ESV [r = 0.67, p < 0.001]). In addition, 6mwt was associated with LA Ee (r = 0.47, p = 0.005), LV GCS (r = 0.45, p = 0.026) and with a statistical trend towards LV GLS (r = -0.38, p = 0.06).

## Discussion

This work comprehensively assessed CMR-derived myocardial morphology and function to characterize its interplay across different entities of HF. Several notable findings should be considered: [1] CMR imaging detects different HF patterns in subgroups with varying cardiac affection. (2) HFpEF is characterized by an impaired atrioventricular interplay accompanied by deteriorated LA function determining the severity of HF and exercise capacity. (3) In HFmrEF patients an increased LA boosterpump function primarily counteracts HF symptoms by maintaining normal LA volumes and LA reservoir function as a counterpart to early ventricular dysfunction. (4) CMR-derived metrics in HFrEF patients show combined LA and LV volume expansions subsequently demonstrating holistic impairment of global myocardial function.

### Diastolic dysfunction

Beyond preserved LVEF, CMR-derived imaging parameters facilitate early detection of LV dysfunction in HFpEF (6). In particular, atrial functional alterations often precede ventricular changes and consequently assuming critical significance in the diagnostic pathway of HFpEF [28]. 

Our work documented significantly increased LACI in HFpEF patients, indicating a disproportional enlargement of LA compared to LV volumes in these patients. Furthermore, patients with HFpEF showed significant impairments in LA LAS, LA reservoir and conduit function. Since the LA is directly exposed to LV pressure especially during enddiastole, LA volume measurements can be regarded as surrogate parameters for LV diastolic function and represent a barometer of LV filling pressures [29]. Furthermore, impaired LA reservoir and conduit function are even considered as most sensitive parameters for diastolic dysfunction that have been demonstrated to precede LA geometric changes [30]. While there may appear to be similarities in atrial failure between diastolic and systolic dysfunction initially, fundamental differences exist between these phenotypes with HFpEF patients being notably more vulnerable e.g. to preload reduction [31]. Consistent with these observations, especially a distortion of atrioventricular coupling primarily originating from the LA (indicated by an increased LACI) emerged as unique feature of diastolic dysfunction in our study. This underscores the high diagnostic accuracy of this parameter for HFpEF, effectively distinguish it from other HF entities [32]. Furthermore, correlations of LA Ee, LV EDV and LACI with NT-proBNP underline the pivotal role of failing atrial function and abnormalities in atrioventricular interplay in determining the extent of HFpEF compared to left ventricular dysfunction [33]. Likewise, the same CMR-derived parameters showed close associations with 6mwt in our study suggesting impaired early ventricular filling is the primary mechanism underlying HFpEF, leading to compromised oxygen uptake and subsequently diminished exercise capacity [34].

However, the exact pathomechanisms of HFpEF are still not fully understood at present. One might speculate whether and to what extend atrial failure is a consequence of ventricular dysfunction or if a primary intrinsic atrial myopathy might lead to subsequent failure [35]. In this context, an increased passive myocardial stiffness and/or slowed myocytal relaxation resulting in an impaired preload reserve in HFpEF might explain different responses of the LA in these patients compared to those with systolic dysfunction [36]. The trend towards numerically increased T1 values compared to healthy controls in our study further highlights the potential influence and contribution of myocardial fibrosis to cardiac stiffness and diastolic dysfunction [37]. Recently, even among HFpEF patients, further stratification according to LVEF above and below 60% has been proposed, identifying fundamentally different sub-phenotypes of HFpEF with different hemodynamic responses on exercise and suggesting diverse pathomechanisms within this HF entity [38].

Importantly, there is mounting evidence highlighting the essential prognostic value of CMR-derived parameters in HFpEF patients [39]. LA strain assessment has emerged as a pivotal tool for early detection of atrial dysfunction that occurs only during exercise stress offering useful diagnostic and prognostic capabilities [40, 41]. Consequently, a comprehensive CMR-based analysis furnishes crucial insights for non-invasive detection and stratification of diastolic dysfunction facilitating precise risk profiling and potentially guiding targeted therapies in the future [42].

### Systolic dysfunction

Amongst patients suffering from systolic dysfunction, LA function was altered according to the severity of LV systolic failure. In HFmrEF patients LA boosterpump function compensated for LV failure and maintained LA reservoir function. Further deterioration in HFrEF resulted in a holistic impairment of LA function and volume enlargement. Once again it is intriguing to explore, whether these findings depict important processes of physiological Frank-Starling mechanism or if the reported metrics reflect intrinsic atrial failure [43]. At first, systolic LV impairment might be counterbalanced by an augmented active LA contraction and a preserved LA reservoir function due to initially heightened atrial contraction forces following atrial myocyte stretch. This compensation mechanism, however, is limited and with progressive HF and LA dilation atrial fiber shortening contractility begin to fail [44]. Therefore, atrial alterations could arise as natural response to failing ventricular performance, as indicated by unchanged LACI values in our study implying relatively equal enlargement of LA and LV volumes. Conversely, an intrinsic atrial myopathy causing distensibility and stiffness of atrial myocytes could be considered, potentially influencing HF processes with varying atrial compensation capabilities [45]. Importantly, in HFmrEF increased atrial boosterpump strain showed significant associations with clinical exercise capacity. Since in advanced stages of systolic HF the decreasing boosterpump function was no longer associated with exercise performance, these results underline findings of prior studies, that identified atrial boosterpump as important modulator of HF and reflecting deteriorating hemodynamics as well as rising filling pressures in progressing systolic dysfunction with additional prognostic information [10, 46]. Moreover, as imaging biomarkers enabling non-invasive assessment of disrupted myocardial structure, native T1, ECV, and LGE were (numerically) increased in HFrEF. It is interesting to speculate whether ventricular dysfunction succeeds fibrotic remodeling processes.

Considering clinical implications, the identification of typical imaging patterns for different stages of HF could optimize future patient management and treatment. This could involve more sensitive HF monitoring and tailored therapeutic strategies based on the phenotype of myocardial dysfunction [47, 48]. For example, in HF patients treated with sacubitril/valsartan, comprehensive strain analyses were previously demonstrated to be accurate indicators of treatment response and prognosis [49]. Furthermore, despite the fact that LVEF is limited by the inability to detect diastolic dysfunction or to reflect segmental abnormalities of myocardial contractility, comprehensive CMR-derived functional analyses might help to detect different cardiomyopathy specific patterns in HF patients [50] or to identify new risk groups by combining diverse CMR metrics. Beyond evaluating functional features of individual cardiac chambers, their inter-play, based on LACI quantification, has been shown to allow gender-specific improved risk prediction models useful for additional risk-reducing strategies [51].

In addition, adverse structural remodeling processes assessed by CMR-derived parameters of tissue composition might further target pharmacological interventions and enable monitoring of therapeutical success of HF treatment and/or antifibrotic agents [52].

As a result, the findings of our study might contribute to a deeper understanding of HF dynamics and the identification of potentially different bio-signatures might even challenge existing HF classifications. These considerations also advocate for the currently shifting paradigms in several areas of cardiac assessment, such as the reevaluation of the simplified concept of ejection fraction and the integration of new cardiac performance parameters applicable to all HF phenotypes [53]. Therefore, CMR imaging could play a key role in future individualized HF management and it is of vital importance to translate these findings and considerations into clinical practice. With an enhanced ability to determine optimal treatment (targets) for each HF patient at the right time, the likelihood of response to treatment and improved outcome could significantly increase [54]. However, the prognostic implications and their potential therapeutic consequences of all hypothesized HF phenotypes in the current work remain unclear. The benefits of more tailored and personalized treatment strategies aligned with individual HF phenotypes need to be thoroughly investigated in future prospective research studies.

### Limitations

Despite comprehensive CMR assessments of different HF phenotypes the study has limitations. The small sample size may introduce bias and limit the generalizability of findings to the wider HF patient population. Therefore, caution is advised when extrapolating results to larger cohorts and future studies including more patients are warranted to validate these findings. Of note, some p-values, although predominantly significant, are borderline and may only indicate a statistical trend after Bonferroni correction, requiring careful interpretation. Furthermore, not all groups were age-matched and patients with systolic HF were categorized solely based on measured LVEF regardless of underlying HF etiology. Moreover, strain values might be influenced by various factors such as comorbidities and individual patient traits suggesting caution in attributing specific pathophysiological HF mechanisms solely to strain values. Therefore, differences in strain values among HF groups may be overinterpreted as distinct phenotypes making thorough validation and accounting for confounders challenging. In addition, a selection bias may exist due to exclusion criteria for CMR. Finally, data on clinical outcome are lacking necessitating further studies to investigate potential prognostic implications of CMR parameters in HF subgroups.

## Conclusions

CMR imaging provides crucial insights into HF pathophysiology by revealing distinct patterns of LA-LV dysfunction and their contribution to cardiac performance across different HF entities. In HFpEF patients, impairments of LA reservoir and conduit function correlate with a volumetric mismatch of LA and LV indicated by an increased LACI, thereby influencing the extend of clinical HF symptoms. In contrast, HFmrEF patients exhibit a delicate balance between declining ventricular function and volumetric enlargement, alongside preserved LA reservoir function and increased booster pump strain values, which ultimately impacts exercise capacity. In HFrEF patients ongoing systolic HF progression can lead to combined atrial and ventricular volumetric enlargement, adverse myocardial remodeling and a comprehensive impairment of all strain parameters. These diverse morphological and functional phenotypes observed in different HF entities underscore the necessity for personalized HF monitoring and treatment strategies based on imaging findings for optimized patient management in the future. More extensive research involving diverse cohorts of HF patients and thorough validation of imaging biomarkers is necessary to confirm and refine these findings.
